# The 2023 Nobel Prize in Chemistry: Quantum dots

**DOI:** 10.1007/s00216-024-05225-9

**Published:** 2024-03-13

**Authors:** K. David Wegner, Ute Resch-Genger

**Affiliations:** Division Biophotonics, Federal Institute for Materials Research and Testing (BAM), Richard-Willstaetter-Straße 11, Berlin, 12489 Germany

**Keywords:** Quantum dot, Synthesis, Photovoltaics, Photocatalysis, Biosensing, Bioimaging

## Abstract

The 2023 Nobel Prize in Chemistry was awarded to Aleksey I. Ekimov (prize share 1/3), Louis E. Brus (prize share 1/3), and Moungi G. Bawendi (prize share 1/3) for groundbreaking inventions in the field of nanotechnology, i.e., for the discovery and synthesis of semiconductor nanocrystals, also termed quantum dots, that exhibit size-dependent physicochemical properties enabled by quantum size effects. This feature article summarizes the main milestones of the discoveries and developments of quantum dots that paved the road to their versatile applications in solid-state lighting, display technology, energy conversion, medical diagnostics, bioimaging, and image-guided surgery.

## Introduction

Many applications of photonic technologies in the material and life sciences require tools that can convert absorbed photons into emitted ones in the ultraviolet (UV), visible (Vis), near-infrared (NIR), and short-wave infrared (SWIR) region of the electromagnetic spectrum. In this regard, quantum dots (QDs) possess unique optoelectronic properties as their size determines their absorbance and photoluminescence (PL) spectra. Furthermore, they exhibit large extinction coefficients and high PL quantum yields (PL QY). In combination with their small nanometer size, QDs became important tools for many research fields and an excellent example for the huge application potential of nanotechnology. In 2023, the long-expected Nobel Prize in Chemistry 2023 was awarded for the discovery and development of synthetical procedures to obtain colloidally stable QDs.

Although it had long been known that nanoparticles can theoretically show quantum phenomena such as quantum size effects (QSE) and size-dependent physicochemical properties, practical and applicable benefits from this knowledge had been long questioned. In the early 1980s, however, Aleksey Ekimov developed a growth technique for semiconductor microcrystals in a glassy dielectric matrix utilizing a diffusion-controlled phase decomposition method of over-saturated solid solutions that permitted to vary the size of the grown nanoparticles in a controlled manner from some tens to thousands of angstroms. Thereby, size-dependent absorption spectra were observed for semiconductor nanocrystals (NCs) composed of atoms from groups I–VII and II–VI of the periodic table, *e.g.*, CuCl and CdS, that significantly differed from those of the respective bulk material [1–3]. This finding was ascribed by Aleksey Ekimov and Alexander Efros to the three-dimensional space quantization of the bound electron–hole pair (exciton) formed by light absorption in these tiny nanoparticles [2].

At about the same time, Louis Brus could demonstrate size-dependent quantum effects in colloidal nanoparticles composed of CdS and ZnS and a size-dependent evolution of the absorption and emission properties from molecular to bulk semiconductor properties [4, 5]. These findings, together with the work of Armin Henglein [6–9] (who died in 2012) on the preparation of ZnS, CdS, CdSe, CdTe, Cd_3_P_2_, Zn_3_P_2_, and PbS nanoparticles with sizes < 10 nm in the early and mid-eighties, laid the foundation for the research field of colloidal QDs. In the beginning, the aqueous syntheses employed for these simple core-only QDs provided moderately emissive QDs with size-dependent absorption and emission spectra and a relatively broad size distribution compared to nowadays QDs. This enabled the first fundamental photophysical and photochemical studies on QDs and the exploration of quantum phenomena such as excitonic transitions in solution at room temperature with broadly accessible and inexpensive instrumentation such as spectrophotometers and spectrofluorometers. These first studies already demonstrated the importance of QD surface chemistry for their optical properties, particularly their PL QY, which is a measure for the efficiency of the conversion of absorbed into emitted photons and can provide a hint for the occurrence of QD concentration-dependent ligand desorption/adsorption equilibria affecting QD stability and dissolution as discussed later [9, 10]. Following the experimental discovery of QSE in colloidal nanoparticles, Louis Brus also presented a model describing the effect of particle size on electron and hole redox potentials for surface chemical reactions. Thereby, using an effective mass approximation and a spherical model potential, the occurrence of QSE could be predicted in 1983 for QDs with sizes below about 5 nm [11].

The broad interest in continuously improving the properties of QDs for advancing their early recognized industrial potential triggered the search for synthetic strategies, which could provide a better size and surface control. In 1993, Moungi Bawendi reported the synthesis of high-quality CdS, CdSe, and CdTe QDs with sizes from 1.2 to 11.5 nm and a high degree of monodispersity, which was reflected by sharp absorption features and a strong band gap luminescence tunable with particle size and choice of material [12]. This synthesis relied on the pyrolysis of organometallic reagents by injection into a hot coordinating solvent, as detailed later, which provided a temporally discrete nucleation and allowed the controlled growth of the resulting nanocrystals. A narrower size distribution of the prepared QDs was then achieved by size selective precipitation of the nanocrystals. This revolutionized QD synthesis and the high quality of this second generation of QDs paved the road for the exploitation of QDs for photonic, biomedical, and renewable energy applications. Thereby, engineered core/shell semiconductor nanocrystals with precisely designed and tailor-made particle architectures and surface chemistries and unique physicochemical and optoelectronic properties became available as novel photonic tools for material and life science applications and consumer products.

## Quantum size effects (QSE) and the design of different QD structures

**Fig. 1:**
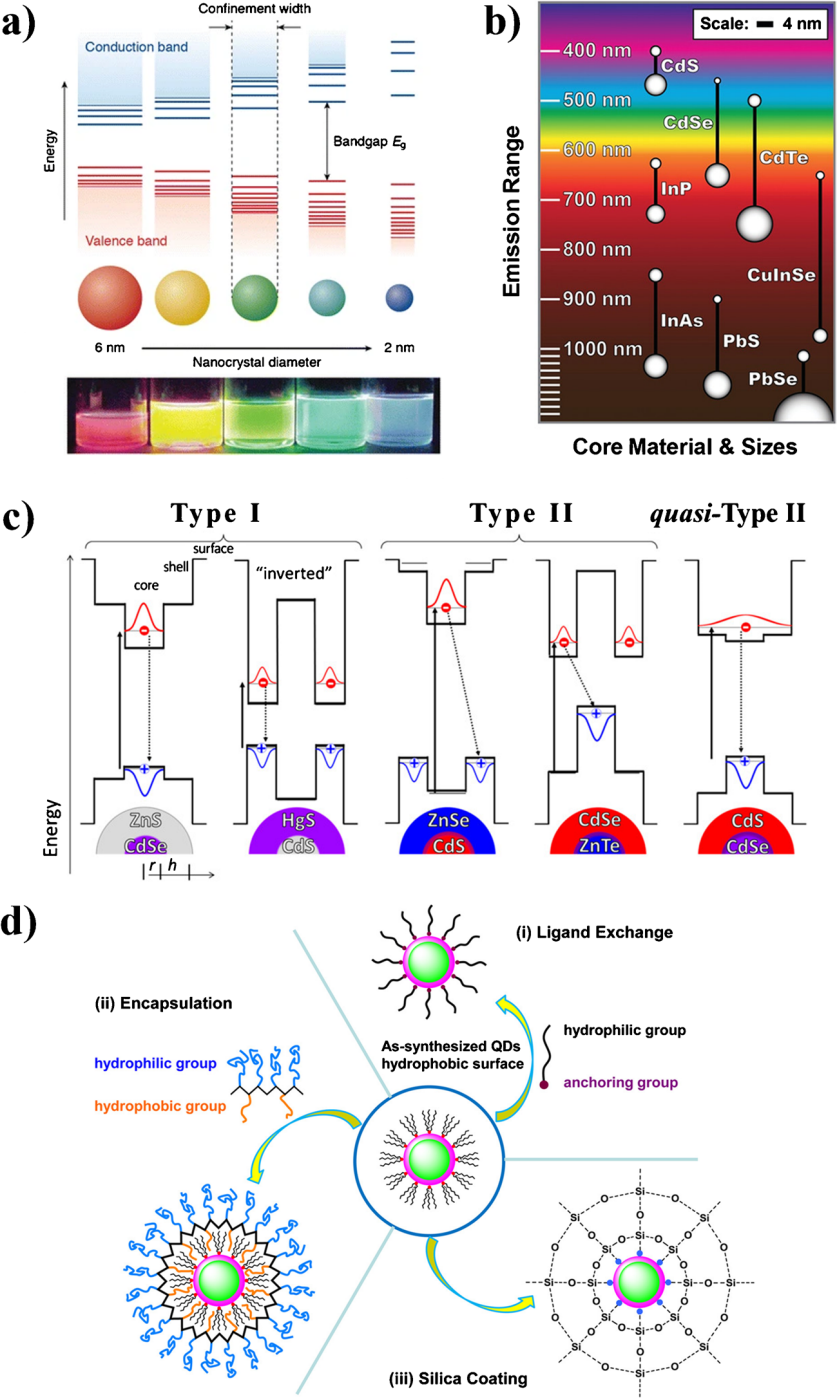
**a** Schematic representation of the quantum size effect (top) and a photograph of five colloidal CdSe QD solutions with different sizes (bottom). Adapted with permission from ref. [17] used under the CC 4.0 license. **b** Photoluminescence (PL) emission wavelength ranges covered by different QD materials. Reprinted with permission from ref. [18]. Copyright 2011 American Chemical Society. **c** Illustration of band gap engineering of core/shell heterostructures made from cores and shells of different composition and band gap, whereby the exciton can be either confined in the core (type I structure), or where the electrons or holes can be located in the shell (type II structure), or where the exciton is localized in the shell (inverse type I structure). Reprinted with permission from ref. [19]. Copyright 2016 American Chemical Society. **d** Schematic presentation of typical strategies of rendering hydrophobic QD water-soluble either by ligand exchange, encapsulation, or silica coating. Reprinted with permission from ref. [20]. Copyright 2016 American Chemical Society

While the physicochemical properties of inorganic materials are largely determined by their constituting atoms or elements, the shrinking of matter to nano-dimensions opens the door for quantum phenomena governed by their size and hence to physicochemical properties, which differ from those of the respective bulk materials. The observation of QSE in semiconductors [13], which are responsible for the fascinating size tunable absorption spectra and luminescence colors of QDs, depends on the semiconductor-specific size of the radius of the exciton, a bound state formed by an electron (e) and a hole (h) that are attracted to each other by Coulomb forces. These charge carriers are created in QDs by absorption of light with a wavelength exceeding the optical band gap (*E*_g_). The size of the exciton or the so-called Bohr radius (R) depends on the dielectric constant and the effective masses of the electron (*m*_e_*) and hole (*m*_h_*) of the semiconductor material and can be between 1 and 10 nm. For QD sizes below the exciton Bohr radius, the energy levels of the valence and conduction band start to split into discrete excitonic states and shift to higher energies, resulting in an increase in *E*_g_ as depicted in Fig. 1a, which is accompanied by a shift of QD absorption and PL to higher energies or shorter wavelengths. As proposed by Louis Brus, the relation between QD size and QD band gap can be described by the particle in the box model; see Eq. 1 [14], where ℏ is the Planck constant:

1 $${E}_{g(QD)}={E}_{g(bulk)}+\frac{{\hslash }^{2}{\pi }^{2}}{2{R}^{2}}\left[\frac{1}{{m}_{e}^{*}}+\frac{1}{{m}_{h}^{*}}\right]$$

Spherical QDs exhibit a size confinement in three dimensions, but the QSE can also occur in two or one dimensions leading to nanostructures referred to as quantum wires and quantum wells. Thus, QDs of different compositions and sizes can cover nearly the whole electromagnetic spectrum ranging from the UV to the NIR (see Fig. 1b). As size-dependent changes in energy-level positions affect also the reduction and oxidation potentials of the charge carriers, QDs are interesting materials for photocatalysis, *e.g.*, for water splitting and hydrogen production [15].

In contrast to the particle in a box principle, a QD does not possess an infinite potential barrier for the confinement of the exciton. The facetted lattice structure abruptly terminates at the QD surface and can lead to localized “trap” states deteriorating the QD’s optoelectronic properties by opening and promoting non-radiative depopulation pathways for excitons formed upon light absorption. To optimize the optical properties and QD stability against photodegradation, the surface of the QD core is often passivated with a shell of a second semiconductor with a higher band gap yielding core/shell heterostructures. Such core/shell systems exhibit strongly improved PL QY values and are less sensitive to changes in the local environment of the QD [16]. Band gap engineering of the core and shell can provide control of the spatial location of the charge carriers in the QD, yielding QD heterostructures where the electron, the hole, or both charge carriers are localized outside of the QD core (see Fig. 1c). A typical example for a type I structure is CdSe/ZnS core/shell QDs where the wider band gap of the ZnS shell favors the confinement of the exciton in the core.

In type II heterostructures, like ZnTe/CdSe and CdS/ZnSe QDs, the electron and hole are localized in the shell and the core or *vice versa* [19]. This design, which facilitates charge separation, is advantageous for applications in photocatalysis and photovoltaics. For reverse type I heterostructures such as ZnSe/CdSe or CdS/HgS QDs, where the charge carriers are localized within the shell, the shell thickness determines the spectral position of the absorption maxima and emission band [16, 21].

Application-relevant physicochemical properties of QDs such as dispersibility and colloidal stability are determined by organic surface ligands. These surface ligands also play an important role in the synthesis of QDs, *i.e.*, nucleation (seed formation) and nanocrystal growth described in the following section. Ligands used for QD synthesis can be divided in hydrophilic ligands for QD synthesis in aqueous solution such as mercaptopropionic acid (MPA), thioglycolic acid (TGA), and glutathione (GSH) and hydrophobic ligands required for the synthesis in organic solvents like oleylamine or oleic acid [22]. These ligands are coordinatively bound to the QD surface atoms and can be removed from the QD surface upon dilution of the QD dispersion, depending on the binding strength of the respective mono-, bi-, or multidentate ligand, resulting in QD concentration-dependent ligand adsorption/desorption equilibria and a possible dependence of PL QY on QD concentration [23, 24]. To adapt the hydrophobicity/hydrophilicity of the QD ligand shell to the desired application, post-synthetic methods for surface modification have been developed; see Fig. 1d, particularly for biosensing and bioimaging applications [18]. This includes the substitution of hydrophobic for hydrophilic ligands (ligand exchange) to render initially hydrophobic QDs water dispersible, which involves, however, the breakage of the ligand–surface atom bonds and can favor the formation of PL QY-deteriorating defect states [25]. The interest in QD life sciences applications in aqueous environments also triggered the design of monomeric and polymeric ligands that strongly coordinate to the QD surface in bioanalytically and biologically relevant media and provide sufficient stability, as systematically explored, *e.g.*, by the group of Hedi Mattoussi [26]. Alternatively, polymer ligands combined with micellar encapsulation and cross-linking of the ligand shell can be used. This prevents ligand desorption but increases the size of the resulting QDs to several 10 nanometers [27]. Also silanization (silica shelling) has been explored that also avoids ligand removal but is still challenging, particularly with respect to the PL QY preservation [20].

## The milestones of QD synthesis

Modern QDs are associated with high-quality optical properties, such as very narrow emission bands, high PL QYs close to unity, and a high photostability. Such nanomaterials can be only achieved with stringent control of the chosen synthesis parameters, chemical precursors, and ligands. The therefore required knowledge has been developed over the last decades [28]. This emphasizes the close relationship between the development of suitable synthesis strategies, QD functionality, and QD applications.

**Fig. 2:**
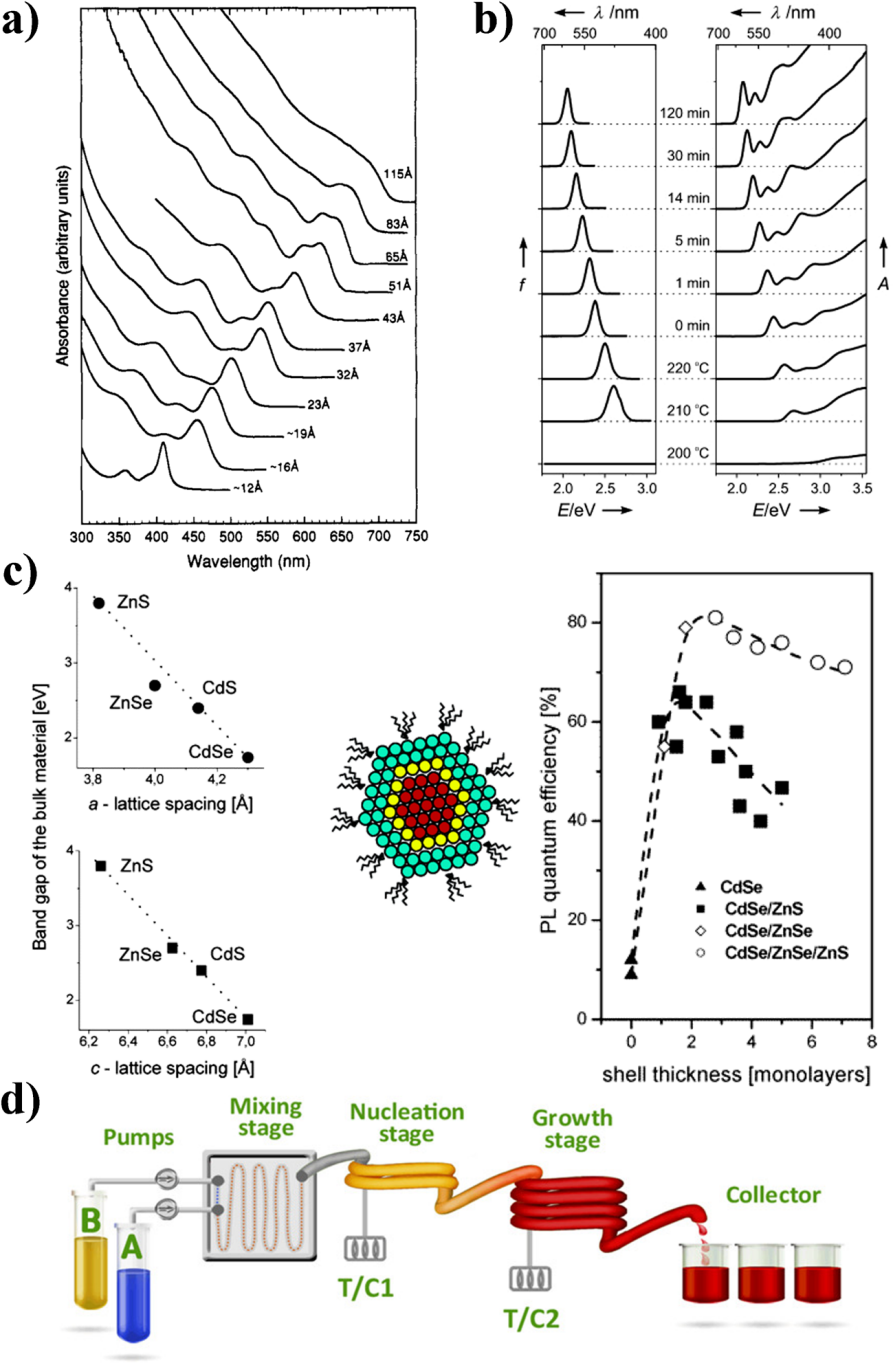
**a** Absorption spectra of CdSe NCs dispersed in hexane with sizes ranging from 1.2 to 11.5 nm. Reprinted with permission from ref. [12]. Copyright 1993 American Chemical Society. **b** Temporal evolution of the PL (left) and the absorption spectra (right) of CdSe QDs during synthesis. Reprinted with permission from ref. [29]. Copyright 2005 Wiley-VCH Verlag GmbH. **c** Relationship between the band gap energy (*E*_g_) and the lattice parameter of bulk wurtzite CdSe, ZnSe, CdS, and ZnS (left) and PL QY values of CdSe and CdSe/ZnS, CdSe/ZnSe, and CdSe/ZnSe/ZnS core/shell and core/shell/shell heterostructures (right). The schematic drawing of the QD in the middle presents the CdSe core (red), ZnSe lattice adapter shell (yellow), and the ZnS outer shell (turquoise). Adapted with permission from ref. [31]. Copyright 2004 American Chemical Society. **d** Schematic presentation of a continuous flow synthesis setup. This setup consists of two syringe pumps transporting the precursors into a mixing stage where both precursors are homogenized before entering the first heating stage where the nucleation occurs. The second heating stage has a lower temperature to ensure no further nucleation and only homogeneous growth. Adapted with permission from ref. [32] Copyright 2013 American Chemical Society

At the beginning of the QD research in the 1980s, the first core-only QDs such as ZnS, CdS, CdSe, CdTe, Cd_3_P_2_, Zn_3_P_2_, and PbS QDs were synthesized by the groups of Louis Brus [4, 5] and Armin Henglein [6–9] in aqueous solution at room temperature. Therefore, commonly an inorganic salt such as Cd(ClO_4_)_2_ as the metal ion source and an inorganic salt like Na_2_S or a gaseous precursor of the respective anion like H_2_S, H_2_Se, and H_2_Te or a phosphine was used together with a stabilizer such as a styrene/maleic anhydride copolymer or a polyphosphate. These aqueous syntheses provided moderate and sometimes even strongly emissive core-only QDs of different size, which were suitable for first fundamental studies of QD photophysics and photochemistry. These QDs were, however, not as monodisperse as modern QDs, and their PL QY were mostly < 50% or less. A major breakthrough of colloidal QD synthesis was realized in 1993 when Christopher Murray, David Norris, and Moungi Bawendi demonstrated the preparation of cadmium chalcogenides using a hot-injection method, which enabled a high level of size control and paved the road to highly crystalline QDs with low defect densities and superior optical properties (see Fig. 2a) [12]. This method relies on the rapid injection of the QD precursors, Cd-TOP, and TOP-Se, into a mixture of hot coordinating solvents such as trioctylphosphine (TOP) and trioctylphosphine oxide (TOPO) at a high temperature of at least 200°C to ensure the rapid decomposition of the precursors and their transformation into monomers, triggering the nucleation of the nanocrystals. The separation of the thereby initiated homogeneous and burst-like occurring nucleation process from the slow and controlled growth of the nuclei, caused by the subsequent depletion of the reagents, prevents further nucleation and yields a narrow size distribution of the QDs. Challenging can be here, however, the upscaling of QD batch size desired for producing larger QD quantities for industrial applications. This triggered the development of other synthetic methods. The most relevant example presents the heat-up method first shown by Charles Cao *et al*., which relies on the continuous heating of the QD precursors in the presence of surface capping ligands (see Fig. 2b). Here, the considerable changes of precursor reactivity with increasing temperature allow for homogeneous nucleation events and continuous growth [29].

Although both hot-injection and heat-up method provided an improved control of QD size and monodispersity, the optical properties of the resulting QDs were still not sufficient for applications requiring stable QDs with close-to-unity PL QYs such as energy conversion, solid-state lighting, and display technology. This changed when synthetic procedures were developed that allowed the controlled sequential growth of core/shell QD heterostructures as shown in Fig. 1c. The milestone for the preparation of such core/shell heterostructures was the development of the successive ion layer adsorption and reaction (SILAR) by Xiaogang Peng, which was first utilized to precisely control the CdS shell thickness on CdSe core QDs [30]. Since then, core/shell synthesis procedure has been developed for a large variety of semiconductor combinations to prepare either type I, type II, or inverse type I QD heterostructures.

Another crucial parameter for the design of QD heterostructures with very high PL QY is the lattice mismatch between the crystal structures of the semiconductors forming the core and the shell, which can result in interfacial structural defects and can lead to the formation of PL diminishing defects and trap states. This challenge could be overcome by the development of core/shell/shell structures. Thereby, a thin intermediate layer of a third semiconductor with a lattice constant in between the lattice constants of the core and outer shell material is introduced to compensate for such lattice mismatches and reduce the lattice strain. Typical examples are CdS or ZnSe lattice adapting shells for QDs, which consist of a CdSe core and an outer ZnS shell. This concept of lattice adapter shells introduced by the group of Horst Weller further improved the optical properties and photostability (see Fig. 2c) [31]. These developments paved the road to the vast majority of the current sophisticated core–(multi)shell QDs with precisely controlled core sizes and shell thicknesses composed of different layers of semiconductors of varying *E*_g_ [16, 28, 33].

Another important step for the field of QD research and photonic applications presented the development of a synthesis resulting in elongated nanocrystals, termed nanorods or quantum rods by the group of Paul Alivisatos [34]. Thereby, ligands are utilized that can “block” specific facets of a QD and direct the growth along one direction to form quantum rods. Such rod-shaped NCs possess a linearly polarized PL and reveal an improved brightness compared to spherical QDs. The combination of controlled PL, polarization, and high PL QY makes such nanorods perfect candidates for optical devices such as LEDs. Current industrially applied QDs are often slightly elongated nanostructures and not spherical [35]. Sandrine Ithurria and Benoit Dubertret eventually pushed the boundaries for nanocrystal shape control by the controlled growth of two-dimensional CdSe structures or so-called CdSe nanoplatelets. The optical properties of such nanoplatelets that reveal extremely narrow emission bands at room temperature are controlled by thickness, *i.e.*, the number of monolayers stacked on top of each other. Such nanoplatelets can be meanwhile synthesized with optical properties covering a wide spectral range. In the last years, even more complicated heterostructure evolved like “dot-in-rods” and “nano-bar-bells” or tetrapods as well as complex core–shell, core–crown, and multishell nanoplatelets [36].

The described fundamental synthesis approaches were initially developed for cadmium or lead chalcogenides. As the inherent toxic potential of the constituting heavy metal ions cadmium and lead hampered industrial and clinical applications, at least in Europe, considerable efforts were dedicated to the synthesis of non-heavy metal containing QDs such as InP QDs as well as to ternary or quaternary QDs composed of three or four constituents such as AgInS_2_ (AIS) and CuInS_2_ (CIS) QDs. The synthesis of meanwhile industrially very relevant InP was first reported by Olga Micic in 1994. However, it was Xiaogang Peng who could speed up the synthesis procedure from days to minutes by using a hot-injection approach, here injecting tris(trimethylsilyl) phosphine into a hot indium fatty acid mixture. The heat-up synthesis approach, demonstrated by Liang Li and Peter Reiss in 2008, strongly improved the reproducibility of InP synthesis [37]. The development of synthetic methods for the large-scale production of high-quality core/shell InP QDs with different emission colors and very high PL QY laid the foundation of the current QD display technology.

Despite the huge advances in QD synthesis awarded with the Nobel Prize in Chemistry, the large-scale production of high-quality QDs with a high degree of reproducibility of the optical properties, *i.e.*, the spectral position and spectral width of the PL band and a high PL QY, and a high photostability is still challenging. The mostly used standard batch syntheses in large flasks are not ideal, as small variations in the handling of the precursors, temperature, stirring speed, *etc.* can largely influence the optical properties of the resulting QDs and can lead to into batch-to-batch variations. Possible solutions can be continuous flow syntheses in small tubular reactors that enable the precise adjustment and control of the reaction parameters such as heating/cooling rates and reaction time, due to the improved heat and mass transfer. A scheme of such a setup is shown in Fig. 2d. This technique can allow faster and automated reactions and an efficient optimization of rapid reactions. Promising examples of this approach for the preparation of different QDs including InP/ZnS and AgInS/ZnS QDs have been meanwhile shown [38, 39].

## QD applications

**Fig. 3:**
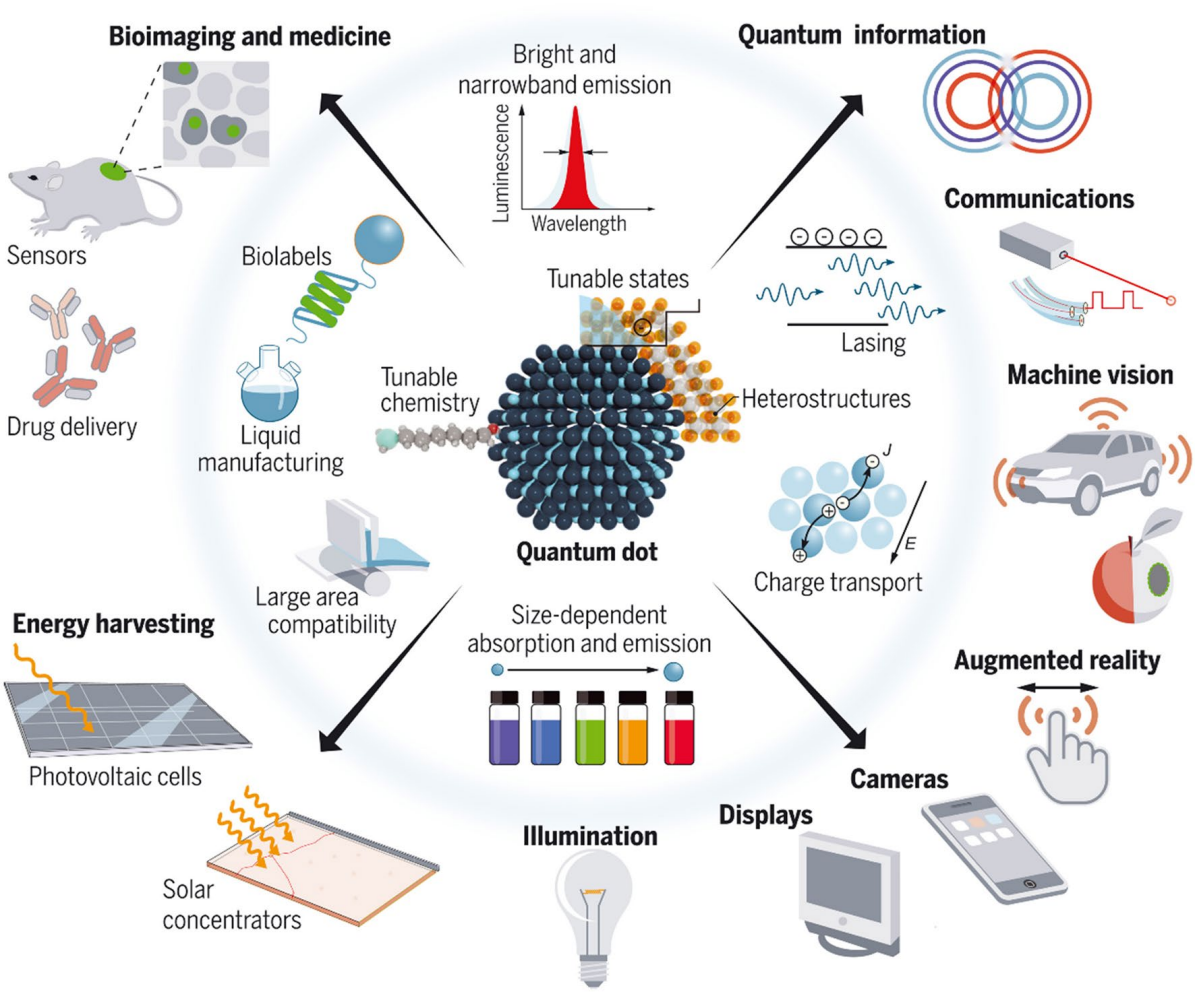
Application fields of QDs exploiting their unique size tunable optical properties such as energy harvesting, conversion, and storage, optoelectronic devices such as displays, detectors, and laser, communication and information technology, bioimaging, biomedical assays, and sensors. From ref. [45]. Reprinted with permission from the American Association for the Advancement of Science (AAAS)

The different application fields of QDs such as display technology, photovoltaics, photocatalysis, biosensing, and bioimaging are highlighted in Fig. 3. Through dedicated synthesis method development highlighted in the previous section, the unique optoelectronic properties of QDs can now be tailor-made for a targeted application. This includes, *e.g.*, the spectral position and width of the narrow Gaussian-like emission bands of binary QDs like CdSe and InP and PL QY tuning to realize values close to unity with dedicated core/shell heterostructures [40].

The first commercial products utilizing QDs were displays for television screens and smartphones, starting with QD-enhanced liquid-crystal-displays (QD-LCDs). In QD-LCD, CdSe and InP core/shell QDs are used for the down conversion of blue light from a InGaN backlight to green and red light. The next generation of displays currently in use relies on QD-based light-emitting-diodes (QLEDs). QLEDs exploit the electroluminescence of QDs and omit the need for a blue backlighting LED. This technology developed by the companies LG and Samsung enabled the fabrication of thinner displays [41]. Other material sciences applications of QDs are, for example, as image sensors. In 2018, QD-based infrared image sensors were first commercialized, which revealed military-grade performance at a fraction of the cost of historical technologies [42]. QDs can also be used as light-harvesting and energy conversion materials in photovoltaic devices, *e.g.*, solar cells and luminescent solar concentrators (LSCs), which are especially interesting for the fabrication of windows with light-harvesting properties for urban architecture [43, 44]. High photon conversion efficiencies (PEC) of >11% of solar cells were, *e.g.*, achieved with PbS QDs.

However, Pb-containing QDs are now considered problematic due to concerns regarding their toxic potential and recent legal restrictions of the usage of lead and cadmium in the European Union [44]. LSCs can be made from less toxic, broad band Vis/NIR emissive ternary QDs with a narrow band gap and a large effective Stokes shift, which prevents photon reabsorption [46].

Life sciences applications of QDs, that were initiated by the groups of Paul Alivisatos and Shuming Nie in 1998 [47, 48], commonly require application-targeted surface engineering by attaching biomolecules such as antibodies, DNA, RNA, or peptides to the QD surface [20, 23]. Applications of QDs as reporters in the life sciences, *e.g.*, in fluorescence assays, multiplexed assays, optical probes for bioimaging studies, and sensors, often exploit the unique broad wavelength excitability of QDs at any wavelength below their band gap. This yields a large effective Stokes shift and allows for the simultaneous excitation of QDs with distinct emission bands using a single excitation wavelength [49, 50]. This renders QDs ideal reporters for color multiplexing, *i.e.*, the simultaneous or parallel measurement of multiple targets and analytes distinguishable based upon their optical properties such as emission color, intensity, or PL lifetime [51, 52]. These features laid the foundation for QD-based assays and sensors utilizing different detection schemes including energy transfer-based signal generation concepts [20]. Other advantageous features for imaging applications present the very large extinction coefficients of most QDs for one-photon and two-photon excitation and their high photostability [52, 53]. The latter is principally ideal for single-particle tracking although QD blinking can present a limitation here [50].

Another emerging application field of QDs in the life sciences presents bioimaging in the near-infrared (NIR) and short-wave infrared (SWIR) at wavelength > 1000 nm, which provide a deeper penetration depth, better resolution, and an improved signal-to-background ratio due to strongly reduced scattering, absorption, and autofluorescence of biological species and tissue in this wavelength region. Typical applications are *in vivo* vascular imaging, *in vivo* biosensing, and theranostics [54]. A unique advantage of QDs are their high PL QY of up to 30%, which are superior to the PL QY of any other molecular or nanoscale NIR/SWIR luminophore [52, 55]. NIR/SWIR-emissive QDs explored include PbS and more promising Pb-free and less toxic InAs as well as Ag_2_S and Ag_2_Se QDs. These applications are closely related to the exploitation of QDs in fluorescence-guided surgery pushed forward by the Bawendi group, which was also highlighted by the Nobel Prize committee. Thereby, QD bioconjugates are used as intraoperative probes to aid surgeons in differentiating between healthy and cancerogenic tissue during cancer resection to reduce the risk of metastases and recurrence of the cancer [56].

QDs can also contribute to the photon-to-chemical energy conversion in photocatalysis reactions. Here, the size-dependent position of their energy levels and redox potentials can be utilized for the design of efficient photocatalytic systems, *e.g.*, for H_2_/O_2_ evolution, CO_2_ reduction, or ammonia generation [57]. In addition, the unique optoelectronic properties of QDs are of interest for quantum computing and quantum communication. QDs can potentially be used as photon and spin qubits, thereby either exploiting their discrete core electron and hole states forming a two-level electronic structure for single-photon emission or by using QDs with precisely controlled spin states caused by defect states and/or dopants introduced into their crystalline structure [45, 58].

## Implications of QD research on analytical chemistry

The advancements in QD synthesis and surface design and their industrial applications described in the previous sections have been flanked and/or enabled by the development of tools for the analytical characterization of these nanostructures. Structure analytical methods closely linked to QD research are, *e.g.*, electron microscopy techniques such as high-resolution transmission electron microscopy (HR-TEM) or scanning transmission electron microscopy (STEM) using high-angle annular dark-field imaging (HAADF). Pulse radiolysis, generating highly reduced or oxidized species and free radicals, combined with fast time-resolved spectroscopic detection, which was employed to monitor the reactions of colloidal QDs with, *e.g.*, hydrated electrons or hydroxyl radicals [59], and the comparison of its results and with data from flash photolysis, that always generates electron–hole pairs, provided the basis for deeper mechanistic insights into QD photophysics in the 1980s. To narrow down the initially broad size distribution of the first generation of QDs prepared in water, size separation methods such as high-performance and size exclusion chromatography were used already during the first QD synthesis [60]. More recently, analytical ultracentrifugation (AUC) has been utilized for multidimensional nanoparticle analysis. AUC with a multiwavelength detector and optical detection can provide information on nanoparticle size, shape, and agglomeration as well as density and optical properties and particle agglomeration in a single experiment without the need for nanoparticle purification [61, 62]. More insights into the coordinatively bound organic ligand shell of QD dispersions could be obtained by liquid NMR methods introduced as a solution NMR toolbox by Zeger Hens [63] as thereby a distinction between free and surface coordinated ligands is possible [64]. In addition to the quantification of surface coordinated ligands, NMR studies are meanwhile utilized to determine the location of ligands at specific nanocrystal facets [63, 65]. These studies contributed to an improved understanding of the QD–ligand interface. The utilization of the advantageous luminescence properties of Vis/NIR/SWIR-emissive QDs for photonic technologies and bioimaging called for the reliable determination of the performance parameter PL QY [66, 67]. This initiated a renaissance of integrating sphere spectroscopy to absolutely measure PL QY of transparent and scattering dispersions as a function of excitation wavelength and triggered the development of measurement techniques for close-to-unity quantum yields [68, 69]. Single-particle photoluminescence spectroscopy, *i.e.*, anti-bunching and intermittency (blinking) studies, contributed to in-depth insight into QD photophysics and their characteristic blinking behavior. These experiments provided information on the ON and OFF times of excitons and the number of QDs from a particle ensemble that are in the ON state per time interval [70].

## Conclusions and recent trends in QD research

The 2023 Nobel Prize in Chemistry recognized the discovery and development of quantum dots (QDs), engineered core/shell semiconductor nanoparticles with precisely designed and tailor-made particle architectures and surface chemistries, that are so tiny that their sizes determine their properties. These quantum size effects (QSE) are responsible for the most fascinating and well-known optical features of QDs, their size controlled and size tunable optical absorption and emission spectra, and their beautiful luminescence colors. These properties, as well as many others, have been meanwhile led to their application in consumer products, now spreading their light from television screens and LEDs. In the life sciences, multi-color emissive QDs contributed to multiplexed detection schemes, assays, optical barcodes, and bioimaging applications.

In the focus of application-driven research in material sciences, nanophotonics, and optoelectronic devices are currently the development of QDs for flexible electronics, tiny sensors, thinner solar cells, and encrypted quantum communication. Other fields of potential commercial use are electrically pumped QD lasers. To make close to perfect QDs even better and lay the basis for many more applications in the material and life sciences, current QD research focuses on further improving syntheses strategies with respect to an even better control of the QD surface and interface, synthesis reproducibility, safe-by-design concepts, and the exploitation of sustainable synthesis approaches, while maintaining QD functionality and performance. For the full exploitation of the very high photoluminescence quantum yields (PL QY) of Cd- and Hg-free NIR/SWIR-emissive QDs such as Ag_2_S and Ag_2_Se QDs for bioimaging applications including the design of intraoperative probes requires the tackling of challenges such as synthesis reproducibility and stability issues. Other recent trends are the synthesis of atomically precise semiconductor nanoclusters for the bottom-up design of the next generation of semiconductor QDs with an extremely narrow size distribution and the monitoring of QD formation processes with 2D-NMR techniques to identify intermediates involved in QD nucleation and growth as well as the study of the QD surface capping ligands [71].

## Declarations

### Disclaimer

The views expressed in this feature article are those of the authors.
